# Experimental and theoretical studies on the molecular properties of ciprofloxacin, norfloxacin, pefloxacin, sparfloxacin, and gatifloxacin in determining bioavailability

**DOI:** 10.1007/s10867-014-9354-z

**Published:** 2014-07-18

**Authors:** E. Kłosińska-Szmurło, F. A. Pluciński, M. Grudzień, K. Betlejewska-Kielak, J. Biernacka, A. P. Mazurek

**Affiliations:** 1 Department of Medicinal Chemistry, Faculty of Pharmacy, Medical University of Warsaw, 1 Banacha Str., 02-097 Warsaw, Poland; 2 National Medicines Institute, 30/34 Chełmska Str., 00-725 Warsaw, Poland

**Keywords:** Fluoroquinolones, Molecular modeling, Partition coefficient, Solubility, Lipophilic properties

## Abstract

The aim of this investigation is to identify, by in silico and in vitro methods, the molecular determinants, e.g., solubility in an aqueous medium and lipophilic properties, which have an effect on the bioavailability of five selected fluoroquinolones. These properties were estimated by analysis of the electrostatic potential pattern and values of free energy of solvation as well as the partition coefficients of the studied compounds. The study is based on theoretical quantum-chemical methods and a simple experimental shake-flask technique with two immiscible phases,* n*-octanol and phosphate buffer. The solvation free energy values of compounds in both environments appeared to be negative. The wide range of electrostatic potential from negative to positive demonstrates the presence of dipole–dipole intermolecular interactions, while the high electron density at various sites indicates the possibility of hydrogen bond formation with solvent molecules. High partition coefficient values, obtained by summing the atomic contributions, did not take various correction factors into account and therefore were not accurate. Theoretical partition coefficient values based on more accurate algorithms, which included these correction factors (fragmental methods), yielded more accurate values. Theoretical methods are useful tools for predicting the bioavailability of fluoroquinolones.

## Introduction

Fluoroquinolones are a group of synthetic antimicrobial agents derived from nalidixic acid that are commonly used to treat acute infections of the genitourinary system and, less frequently, the lower and upper respiratory tract. Their primary mechanism of action is inactivation of bacterial DNA topoisomerase II. They also interact weakly with DNA topoisomerase IV, thus inhibiting replication of bacterial cells and leading to their death. Fluoroquinolones have a broad antibacterial spectrum. First-generation fluoroquinolones are, with a few exceptions (e.g., pipemidic acid), becoming less commonly used because of their poor bioavailability, narrow spectrum of activity, development of microbial resistance, and more side effects than those induced by fluoroquinolones of newer generations. The range of antimicrobial activity exhibited by newer fluoroquinolones includes most Gram-negative bacteria (*Salmonella*,* Shigella*), Gram-positive bacteria (*Streptococci* and* Staphylococci*) and atypical strains such as* Chlamydia*,* Mycoplasma*,* Legionella*,* Pseudomonas aeruginosa*, and* Mycobacterium tuberculosis*. Drugs of the second, third, and fourth generations contain an additional fluorine atom at position 6 of the quinolone core, which affects the pharmacokinetic profile of fluoroquinolones in polar environments and improves their bioavailability and antibacterial activity [1–3].

Diprotic molecules like fluoroquinolones contain acidic and basic groups and, depending on the pH of the aqueous environment, they can occur in four protonated forms: cationic, zwitterionic, neutral, and anionic. The equilibrium between the forms can be shifted by pH changes. At the pH corresponding to the isoelectric point, zwitterions and neutral molecules are present at their highest concentrations. Neutral molecules exhibit the lowest solubility in water and in other polar solvents. However, in non-polar media, such as lipids in the cell membrane, their solubility is higher, so they can easily pass through cell membranes and show good bioavailability. By contrast, zwitterions have a greater affinity for polar environments and penetrate easily through pores in bacterial cell walls, after which they readily bind to bacterial gyrase [4–7].

**Fig. 1:**
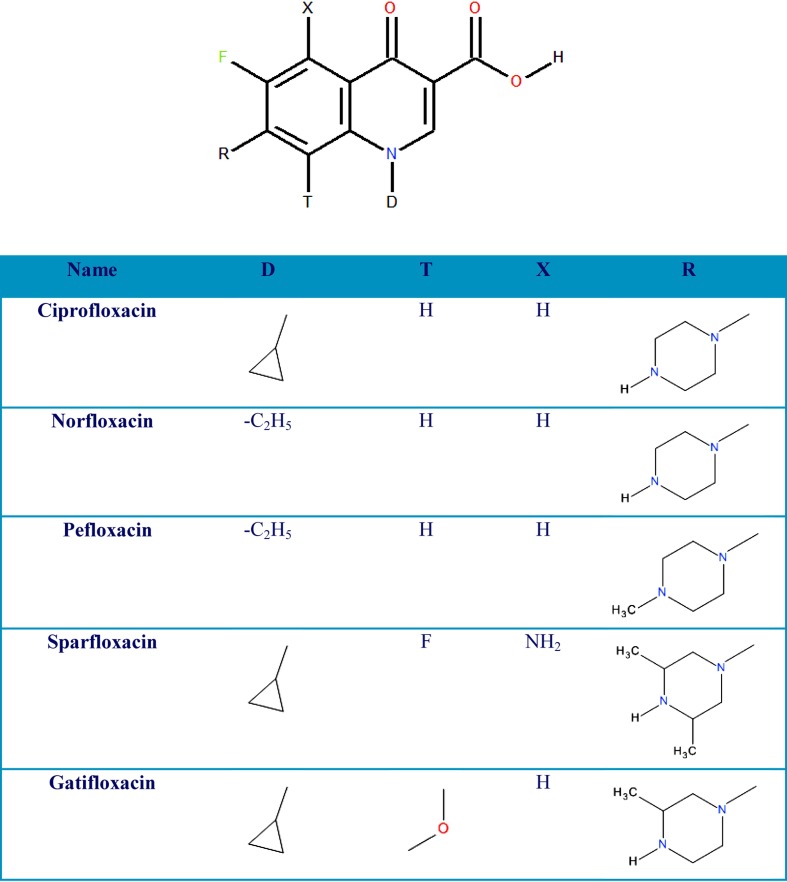
Chemical structures of the six studied fluoroquinolones

The bioavailability of a drug substance is determined by the first and second stage of the drug’s fate in the body (liberation and absorption). Here, the aim of the study is to evaluate the usefulness of simple and inexpensive experimental and theoretical methods to estimate the bioavailability of the studied compounds. We calculated the thermodynamic and electrostatic properties of ciprofloxacin, norfloxacin, and its methylene derivatives pefloxacin, sparfloxacin, and gatifloxacin (Fig. 1). These properties are: the free energy of solvation in water and diethyl ether, the electrostatic potential surface of the molecule, the logarithm of the* n*-octanol/water partition coefficient (P_o/w_) and the logarithm of the apparent *n*-octanol/buffer partition coefficient.

The theoretical method comprised quantum-chemical calculations through which the lipophilicity parameters of fluoroquinolones were determined. For theoretical evaluations of lipophilicity, the following determinants were calculated: the electrostatic potential on the surface of a drug molecule, which, inter alia, indicates the number of electron donor sites as a measure of the electrostatic interactions of the molecules with the solvent and possible formation of hydrogen bonds, the free energy of solvation of the molecule in water or diethyl ether and its partition coefficient between the two media. In drug design, it is the *n*-octanol-water partition coefficient, log P _o/w_, which is commonly used as the standard lipophilicity parameter [8]. P_o/w_ is directly related to the free energy of binding [9, 10]. P_o/w_ is an equilibrium constant for solute transfer between octanol and water, as evidenced by the following equation:

1 $$ \log\ {\mathrm{P}}_{\mathrm{o}/ w}=\hbox{--} \Delta \mathrm{G}{}^{\circ}/2.303\ \mathrm{RT} $$

where T is the absolute temperature, R the gas constant, and ΔG° the binding free energy variation measured under standard conditions (298 K, 1 atm, and 1 M concentration for both reagents and products).

To experimentally determine the apparent partition coefficient of the drug substance, we used the shake-flask technique with two immiscible phases,* n*-octanol and phosphate buffer at pH 6.8. Aqueous buffer was used instead of water as it provided an environment similar to that in the gastrointestinal tract, the site of drug absorption.

## Materials and methods

### Materials

The compounds tested were from Grunenthal GmbH (Aachen, Germany) (ciprofloxacin hydrochloride, norfloxacin, gatifloxacin) and Sigma-Aldrich, US (pefloxacin mesylate, sparfloxacin).* n*-Octanol was purchased from Emplura (Merck, Darmstadt, Germany).

The equipment used included a JASCO 530 UV–Vis spectrophotometer, a mechanical shaker from Janke & Kunkel, IKA Labortechnik, HS 250, a Mettler-Toledo MP 225 model pH meter, R program v. 2.14.2 with R Studio graphic overlay v. 0.95.262, an Intel Core i5 CPU computer with an AMD Radeon HD 6800 series graphic card, Spartan 08 software package, ACD/ChemSketch freeware v.12 and the Virtual Computational Chemistry Laboratory (VCCLAB) on-line software package (ALOGPS 2.1).

### Methods

#### Calculation of thermodynamic parameters

In the first stage of the study, the conformational space was searched for each compound in order to determine the most stable, lowest energy conformers. These calculations were performed by the Monte Carlo method with an implemented MMFF94 force field [11]. The lowest energy conformer was the initial structure in the later geometry optimization stage of the calculations. Next, the free energy of solvation in water and diethyl ether was determined for the most stable conformers of all the compounds by employing the SM8 continuum solvent model and the density functional theory (DFT) method with the gradient corrected using the B3LYP (Beck-Lee-Yang-Parr) functional and the 6-31*G basis set as implemented in the Spartan 08 software package [12]. The free solvation energy was calculated as the difference in binding energy between the molecule in the gas phase and the molecule in solution; more precisely, this difference involves a change in all possible points of the molecular surface interaction when the molecule is in the gas phase, then it is placed in the selected liquid environment. Diethyl ether was selected because its dielectric constant is close to that of* n*-octanol, which is not parameterized within Spartan 08. The electrostatic potential on a surface of equal electron density was calculated using the same functional and basis set as for the geometry optimization, in order to determine the contribution of electron donor and electron acceptor sites into the electrostatic pattern of the molecule. In addition, the value of the electrostatic potential was checked using the 6–31 G** basis set.

#### In silico models

Values of the partition coefficient were calculated using associated neural networks and other combined models to predict hydrophilic and lipophilic properties [13, 14]. These calculations rely on two basic rules: that (1) molecules are cut into fragments or atoms (fragmental methods and atomic-contribution methods or surface areas) and (2) each fragment is then summed up. The fragmental method developed by Rekker and Mannhold shows that this lipophilicity descriptor can be calculated as the sum of the fragment values taking into account certain correction factors. These correction factors can be related to tautomerization effects, dipolar effects, ion effects, proximity effects, electronic interactions within one fragment, conjugated multi-hetero atomic effects, steric effects, H-bonding interactions, and hydrophobic constants [15, 16].

According to this method, the log P value was calculated according to the equation:

2 $$ \log \kern.3em \mathrm{P}=\varSigma {\mathrm{k}}_1{\mathrm{f}}_1+\varSigma {\mathrm{l}}_2{\mathrm{F}}_2 $$

where k is the number of repeats of fragment f of type 1, and l is the number of repeats of correction factor F of type 2 [17].

The fragment values include averaged contributions of simple fragments derived from a large database of experimentally measured log P values. The ACD/log P, AB/log P algorithms included in ACD/ChemSketch freeware v.12 I-Lab 2.0 (v 5.0.0.184) were used according to the fragmentation rules. MiLog P from http://www.molinspiration.com/services/logp.html takes into account charge interactions as well. A similar method, AC/log P, from the VCCLAB on-line calculator, is based on Hansch and Leo’s approach and takes structure-dependent correction values from their database into account [9, 18]. In the Ghose/Crippen approach, values of log P can be verified with the use of atomic-contribution methods [19].

The method to obtain log P is represented by the following equation:

3 $$ \log \kern.3em \mathrm{P}=\varSigma {\mathrm{m}}_1{\mathrm{n}}_1 $$

where m_1_ is the number of atoms of type 1 and n_1_ is the contribution of an atom of type 1.

These algorithms, among others, are accessible in the VCCLAB online software package (ALOGPS 2.1) and implemented in Spartan 08 software and were used in our study. It is worth mentioning that atomic-contribution methods do not take correction factors into account [16, 20–23].

#### Experimental determination of log P_o/w_

Determinations were performed using the shake-flask technique [24]. The temperature of the separation media was controlled and kept at 24 ºC. The amount of the drug substance used to determine the partition coefficient in* n*-octanol/buffer was fixed in such a way as not to exceed a concentration of 0.01 mol/l. Each substance was dissolved in 0.1 M phosphate buffer at pH 7.4 and then the pH of the solution was adjusted to pH 6.8 with 1 M HCl. After dilution of the samples, concentration vs. UV absorbance calibration curves were drawn for five different concentrations of each compound. The absorbance was measured at a specific UV wavelength λ_max_ for each compound. For each test substance, the Pearson linear correlation coefficient was not less than 0.9993, whereas the* p* values were in the range of 1.35 × 10^−6^ to 7.55 × 10^−6^ [25]. Before the shake-flask experiments, the buffered samples were pre-saturated with 0.1 M * n*-octanol. The samples were shaken for a few hours in a flask and then separated in a separator and, after reaching the steady state, the aqueous phase was centrifuged at 650 × *g*. The concentration of the compound in the aqueous phase was determined based on the calibration curve. Then, the concentration of the compound in *n*-octanol and the apparent partition coefficient were calculated. Each of the values calculated is an average of three parallel measurements.

## Results

### Thermodynamic potential of solvation and electrostatic potential on the surfaces of the molecules

In the first stage, using the DFT method with the gradient corrected using the B3LYP functional and the 6-31*G basis set as implemented in the Spartan software package, it was decided to analyze the values of the calculated free energy of solvation. As shown in Table 1, the solvation free energy values of the compounds in water and the organic solvent were largely negative. Thus, values for cationic forms of ciprofloxacin, norfloxacin, and pefloxacin were determined, as well as zwitterionic/neutral forms for ciprofloxacin, pefloxacin, norfloxacin, sparfloxacin, and gatifloxacin. The negative free energy of solvation in diethyl ether, representing the lipophilic solvent, was higher than in water. Also, the negative free energy of solvation of the cationic forms in diethyl ether was higher than the energy of solvation of the zwitterionic/neutral forms. For ciprofloxacin, the difference between the energy of solvation in water and diethyl ether was higher compared to the other compounds.

**Table 1:** Thermodynamic and electrostatic potential

The test substance	Free energy of solvation (kcal/mol)		Electrostatic potential (kcal/mol)
	Water	Diethyl ether	
Ciprofloxacin	–61.07*	–76.86*	(−)20.55* – (+)165.84
Norfloxacin	−10.64 –59.97*	−17.45 –68.75*	(−)64.60 – (+)49.80 (−)20.49* – (+)162.69
Pefloxacin	−8.51 –52.87*	−17.04 –62.37*	(−)63.15 – (+)50.30 (−)22.08* – (+)156.92
Sparfloxacin	−13.43	−16.01	(−)71.82 – (+)50.73
Gatifloxacin	−7.83	−17.69	(−)56.98 – (+)38.59

^*^Cationic form

**Fig. 2:**
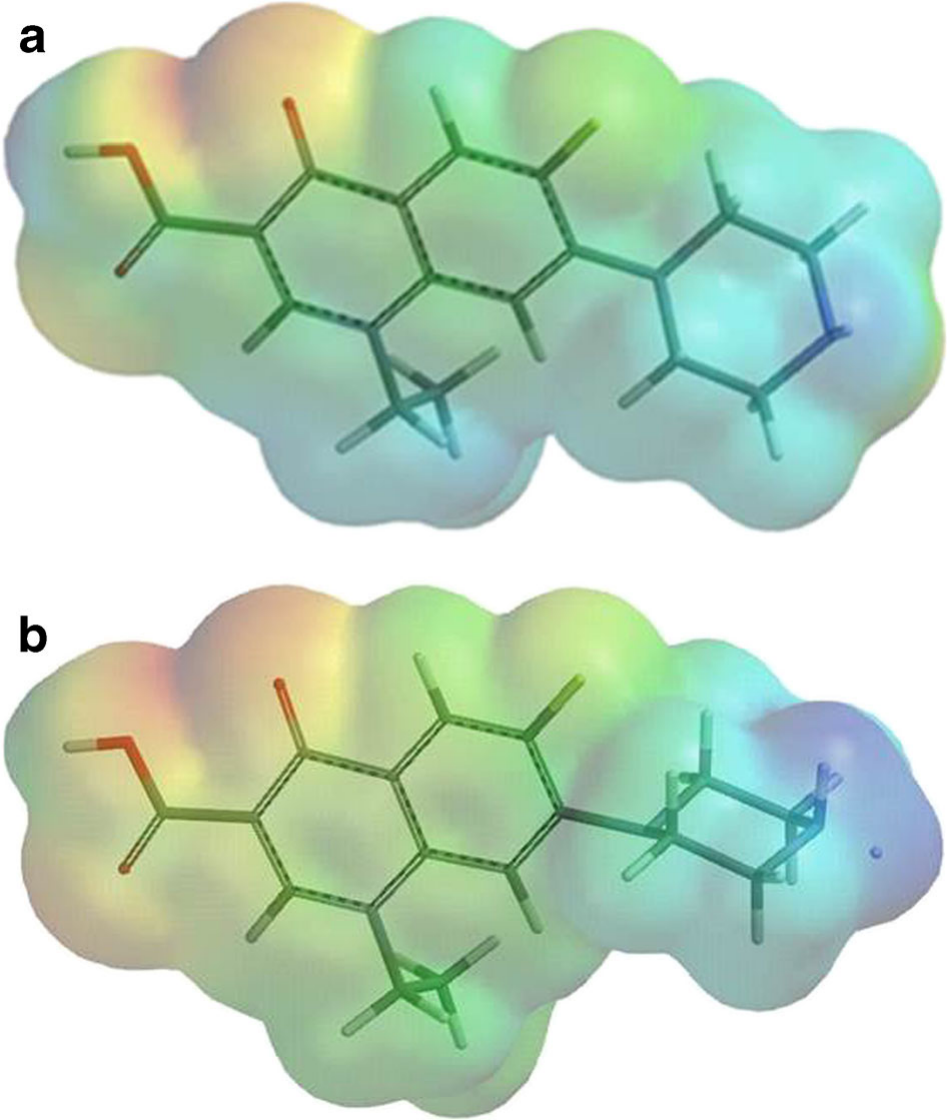
**a** The optimized geometry and electrostatic potential pattern on the surface of norfloxacin (*red* negative, high electron density;* blue* positive area, low electron density). **b** N-protonated form of norfloxacin (*red* negative, high electron density;* blue* positive area, low electron density)

Figure 2a and b show an example of an optimized model of the molecule norfloxacin and its cationic form. Values of electrostatic potential show a wide range of electrostatic potential around the studied molecule. As shown in Table 1, the electrostatic potential pattern was changed significantly after protonation of the nitrogen on the piperazine moiety. In the presence of the solvent, the values of the pefloxacin electrostatic potential change were calculated as follows: φE_water_ = (−) 59.62 – (+) 41.90, φE_gas phase_ = (−) 63.15 - (+) 50.30, and φE_diethyl ether_ = (−) 64.02 – (+)52.24, respectively. A similar dependence, i.e., decreasing in water and increasing in diethyl ether compared to the gas phase, occurred in the case of other molecules. The electrostatic potential was also calculated based on a more advanced basis set, 6-31G**. For example, the electrostatic potential values of pefloxacin in the gas phase were (−) 63.15 - (+) 50.30 for the 6–31 G* basis set and (−) 63.21 - (+) 50.41 for the 6–31 G** basis set.

### Theoretical and experimental partition coefficient

In the next stage of the study, the logarithm of the *n*-octanol/water partition coefficient was calculated using various in silico models, such as ACD/log P, ALOGP, MLOGP, AB/log P, AC/log P, or miLog P (Table 2) and the experimental shake-flask method (Table 3). The experimentally obtained pefloxacin partition coefficient value was greater than zero and showed the good properties of this lipophilic compound in a physiological medium. The negative values of norfloxacin, ciprofloxacin, sparfloxacin, and gatifloxacin log P indicate a high affinity of the compounds into a polar environment at a given pH (6.8). The theoretical partition coefficient calculated values were fairly different. The results obtained using the fragmentary and atomic contribution methods showed considerable discrepancy.

**Table 2:** Theoretical values of partition coefficients for test compounds

	ACD/log P^a^	ALOGP^b^	MLOGP^b^	AB/log P^c^	AC/log P^b^	miLog P^d^	Log P^e^
Ciprofloxacin	0.65 ± 1.44	1.41 (+1.1)	1.67 (+1.4)	−0.71	0.13 (−0.15)	−0.701	1.39
Norfloxacin	0.82 ± 1.44	1.27 (+2.3)	1.43 (+2.5)	−0.92	−0.06 (+0.97)	−0.691	1.44
Pefloxacin	1.51 ± 1.43	1.8 (+1.53)	1.67 (+1.4)	−0.08	0.22 (−0.05)	−0.095	1.82
Sparfloxacin	1.2 ± 1.57	1.62	2.00	−0.23	0.04	1.628	1.31
Gatifloxacin	1.21 ± 1.54	1.77	1.38	0.01	0.31	−0.036	1.51

^a^Values from ACD/Labs v 5.0.0.184

^b^Values from experimental database VCCLAB (ALOGPS 2.1.)

^c^Values from ACD/ChemSketch v 2.0

^d^miLog P – www.molinspiration.com

^e^log P values from Spartan 08

**Table 3:** Experimental and reference values of log P for tested compounds

The test substance	Experimental and reference values of log P			
	log P_exp.* n*-oct/buf pH=6.8_^a^	log P	log P _pH = isoelectric point_^b^	log P_true_^b^
Ciprofloxacin	−0.55	−1.10 _*n*-oct/buf pH = 7_^c^	−1.07	−0.13
Norfloxacin	−0.92	−1.52 _*n*-oct/buf pH = 7_^c^	−1.07	−0.43
Pefloxacin	0.19	0.26 _*n*-oct/buf pH = 7_^c^	0.37	1.34
Sparfloxacin	−0.11	−0.31 ^d^	−0.09	0.39
Gatifloxacin	−0.80	-	−0.71	0.54

Values from:

^a^Our results

^b^G. Volgyi et al., 2012 [5];

^c^Y. X. Furet, J. Deshusses, J. C. Pechere., 1992 [26];

^d^O. Cramariuc et al., 2012 [27]

## Discussion

The free energy of solvation, as mentioned earlier, is an important parameter that takes into account both the enthalpy and entropy of non-covalent bond formation between a dissolution molecule and its environment, in fact, the interaction that occurs between molecules of the solute and the solvent. The free energy of binding affects both the hydrophilic and lipophilic properties of molecules. Negative values of the free energy of solvation show that the dissolution reaction occurs spontaneously and there is no obstacle to the substance dissolving in the aqueous environment and passing through biological membranes. As in the gastrointestinal tract the pH environment at the site of penetration is below the isoelectric point of the compound [5], it was decided to test the free energy for a possible ionization state at a physiologically relevant pH. The data (Table 1) show that the studied molecules in the case of both cationic and zwitterionic/neutral forms interact more strongly with an organic solvent than with an aqueous solvent. The compounds in which the nitrogen of the piperazine group is protonated provided significantly higher negative free energy values than those with the charge distributed uniformly for both end groups, or those that were neutral.

The electrostatic potential pattern is adequate to explain the interactions between the solvent and the solute [28]. A map of electrostatic potentials provides information on possible solvent molecule distributions around the functional groups of the dissolved molecule (Fig. 2a, b). The pattern of its electrostatic potential field determines the polarity of a compound and predetermines the interactions of the molecule with an aqueous medium. The wide range of electrostatic potential around the studied molecules indicates an interaction with the polar solvent based on the formation of dipole–dipole or dipole-induced dipole type electrostatic interactions (Fig. 2a, b, red and blue areas of the molecule). A number of electron donor sites in the molecule allow the creation of hydrogen bonds with the polar solvent. The range of the positive electrostatic potential around the cationic form indicates the inability to form hydrogen bonds necessary for the interaction of the test substance and water molecules (Table 1). It is true that a more advanced basis set choice may slightly or insignificantly impact the electrostatic potential values. However, the differences in the calculated values of electrostatic potential in the gas phase and in the presence of the solvent are not more important here because the selected model did not exactly describe the dispersion interactions. In the polarizable continuum model, the second shell is more diffused and there is less orientation of the dipoles of the solvent molecules on the dissolved molecule. This model does not take into account specific interactions, such as hydrogen bonding between solute and solvent molecules, but only considers the average effect of solvation, which the Coulomb forces simulate and is affected by dipole-induced dipole interactions.

High log P values, obtained by summing the atomic contributions, showed the imperfections of algorithms that do not take various correction factors into account. It is evident that the correction factors, such as steric (Hansch constant) and electronic (Hammett constant) parameters and atomic hydrophobic contributions should play a key role in theoretical calculations [23, 29]. The theoretical partition coefficient values based on more accurate algorithms, which included the correction factors (fragmental methods) [16, 17] such as AB/log P, AC/log P, or miLog P (Table 2), yielded values only slightly different than the experimentally obtained values in this study or reference values obtained by the authors [30–33]. These values seem to be more comparable, although there are still some notable differences. These differences result from the use of various correction factors, specific to each method. Some approaches develop a purely atom-based procedure, thereby avoiding correction factors [16]. Pefloxacin seems to be strongly lipophilic, while norfloxacin and ciprofloxacin seem to be strongly hydrophilic, despite the large value of negative thermodynamic potential of solvation in the apolar solvent. In contrast, sparfloxacin appears to be moderately lipophilic, which has been confirmed by other authors [29, 31–33]. The lipophilicity of gatifloxacin (the fourth-generation group) is controversial. The difference value of the partition coefficient between norfloxacin and pefloxacin shows that even a small change in the molecule (addition of a methyl group to the nitrogen of the piperazine ring) can greatly change the bioavailability properties of a compound. The discrepancies between log P values can be explained by the changing states of ionization [26]. Even in an environment close to the isoelectric point, the zwitterionic form predominates [34]. Interestingly, the zwitterionic form, despite the high electric field produced by each dipole, passes through the membrane mainly via passive transport [27]. The answers to these considerations require insights into intramolecular and intermolecular interactions. Any change in the charge distribution, formed in the presence of electronegative atoms such as oxygen or nitrogen, raises the dipole moment between the carbon atoms in the molecule; intramolecular hydrogen bonds additionally stabilize the molecule [35]. Interesting conclusions have been put forward by other authors [36]. They claim to have observed that neutral and zwitterionic ciprofloxacin form agglomerates. Neutral molecules were present inside liposomes, where there is a polar environment. However, zwitterions formed stacks in the presence of the hydrophobic part of the membrane through which they pass. The creation of such clusters is due to hydrophobic interactions and hydrogen bonds, and may play an important role in their activity [36, 37]. As noted by Cramariuc et al. [36], if particles are dipoles and have an electric field, then they are able to arrange in an antiparallel fashion, thus reducing the electric field and increasing their activity in an apolar environment. This feature should improve their lipophilicity and thus enable them to pass through the membrane by passive transport. Further studies by these authors have demonstrated that such agglomerates move between the polar and apolar environment by intermolecular transfer of protons, during which the particles do not lose their stability and retain their solvation ability. Other authors [38] have noted that the difference between the theoretical partition coefficients obtained based on calculations as opposed to experimental studies may be due to interactions occurring between the cationic form of solute molecules and the anionic buffer; the formation of such an ion complex may result in these differences.

## Conclusions

Ampholytes, such as fluoroquinolones, make it very difficult to accurately assess their lipophilicity on the basis of theoretical methods that do not address all the thermodynamic phenomena occurring between the molecules and the environment in which the compound is located, as well as intramolecular interactions between the functional groups.

The elimination of the high total charge of the electric field that allows the passage of fluoroquinolone molecules through biological membranes explains the phenomenon of agglomerate formation by zwitterions. The higher negative values of the thermodynamic solvation potential in diethyl ether compared to that in water confirms the good lipophilic properties of these compounds and demonstrates their high intrinsic lipophilicity.

It is worth noting that the methods used in this study can also be used as a convenient tool for initial and rapid substance classification into the four classes of the Biopharmaceutical Classification System and serve to enlarge this system [39, 40]. To date, the WHO has classed only three active substances in this group of drugs, i.e., ciprofloxacin hydrochloride, ofloxacin, and levofloxacin, with ciprofloxacin in the third class and levofloxacin and ofloxacin in the first [41]. The correctness of the classification of ciprofloxacin to the third BCS class seems to confirm the experimentally determined log P_o/w_ and theoretical results obtained only with the algorithms AB/log P and miLog P, as well as the electrostatic potential and free enthalpy of solvation in water, while the value of free solvation enthalpy in diethyl ether provides evidence of its high intrinsic lipophilicity.

Theoretical methods are good tools in the prognosis of the thermodynamic properties of molecules and thus in screening tests to evaluate the liberation and absorption of new therapeutic substances.
